# Safety and performance of the Vienna self-expandable transcatheter aortic valve system: 6-month results of the VIVA first-in-human feasibility study

**DOI:** 10.3389/fcvm.2023.1199047

**Published:** 2023-07-13

**Authors:** Kasparas Briedis, Vaida Mizariene, Egle Rumbinaite, Martynas Jurenas, Ali Aldujeli, Kamilija Briede, Povilas Jakuska, Antanas Jankauskas, Indre Ceponiene, Tadas Lenkutis, Remigijus Zaliunas, Rimantas Benetis

**Affiliations:** ^1^ Department of Cardiology, Lithuanian University of Health Sciences, Kaunas, Lithuania; ^2^ Department of Cardiac, Thoracic and Vascular Surgery, Lithuanian University of Health Sciences, Kaunas, Lithuania; ^3^ Department of Radiology, Lithuanian University of Health Sciences, Kaunas, Lithuania

**Keywords:** TAVI, aortic stenosis, first-in-human (FIH), dry-pericardium, Vienna TAVI, VIVA trial, Vienna aortic valve, pre-mounted TAVI

## Abstract

**Background:**

The novel Vienna TAVI system is repositionable and retrievable, already pre-mounted on the delivery system, eliminating the need for assembly and crimping of the device prior to valve implantation.

**Aims:**

The purpose of this first-in-human feasibility study was to determine the safety, feasibility, clinical and hemodynamic performance of the Vienna TAVI system at 6-month follow-up. (ClinicalTrials.gov identifier NCT04861805).

**Methods:**

This is a prospective, non-randomized, single-arm, single-center, first-stage FIH feasibility study, which is followed by a second-stage pivotal, multicenter, multinational study in symptomatic patients with severe aortic stenosis (SAS). The first-stage FIH study evaluated the safety and feasibility, clinical and hemodynamic performance of the device in 10 patients with SAS based on recommendations by the VARC-2.

**Results:**

All patients were alive at 3-month follow-up. 1 non-cardiovascular mortality was reported 5 months after implantation. There were no new cerebrovascular events, life-threatening bleeding or conduction disturbances observed at 6-month follow-up. The mean AV gradient significantly decreased from 48.7 ± 10.8 to 7.32 ± 2.0 mmHg and mean AVA increased from 0.75 ± 0.18 to 2.16 ± 0.42 cm^2^ (*p* < 0.00001). There was no incidence of moderate or severe total AR observed. In the QoL questionnaires, the patients reported a significant improvement from the baseline 12-KCCQ mean score 58 ± 15 to 76 ± 20. NYHA functional class improved in two patients, remained unchanged in one patient. There was an increase in mean 6-min-walk distance from baseline 285 ± 97 to 347 ± 57 m.

**Conclusions:**

This study demonstrates that using Vienna TAVI system has favourable and sustained 6-month safety and performance outcomes in patients with symptomatic severe aortic stenosis.

## 1. Introduction

Transcatheter aortic valve implantation (TAVI) was initially limited to patients with symptomatic severe aortic stenosis (AS) who were considered to be at prohibitive risk for surgical aortic valve replacement (SAVR) due to their age, health condition, or other factors (1). However, as TAVI has been studied more, it has been shown to be effective in patients with high, intermediate, and even low surgical risks (2–4). Over the past decade, TAVI has been approved across all risk groups, including high, intermediate, and low. This expanded approval has consequently led to an increase in the number of TAVI procedures performed. According to data from 2019, almost twice as many TAVI procedures were performed compared to SAVR in the United States (5). Moreover, it is anticipated that the clinical indications for TAVI will diversify in the forthcoming years, thereby broadening its therapeutic scope to an increased number of patients (6, 7). The development of novel TAVI techniques and technologies is aimed at enhancing safety and efficacy, which could make it a suitable intervention for patients with asymptomatic severe aortic stenosis, symptomatic moderate aortic stenosis, severe aortic regurgitation, as well as younger patients who were previously considered less likely candidates for TAVI (6, 7).

While TAVI outcomes have significantly improved due to better patient selection, increasing user experience, and iterations in device design, important issues remain. Clinical challenges where further advances would be desirable include the occurrence of major procedural complications, stroke, paravalvular aortic regurgitation, vascular complications and need for new permanent pacemaker implantation (8).

## 2. Methods

### 2.1. Study device

**Figure 1:**
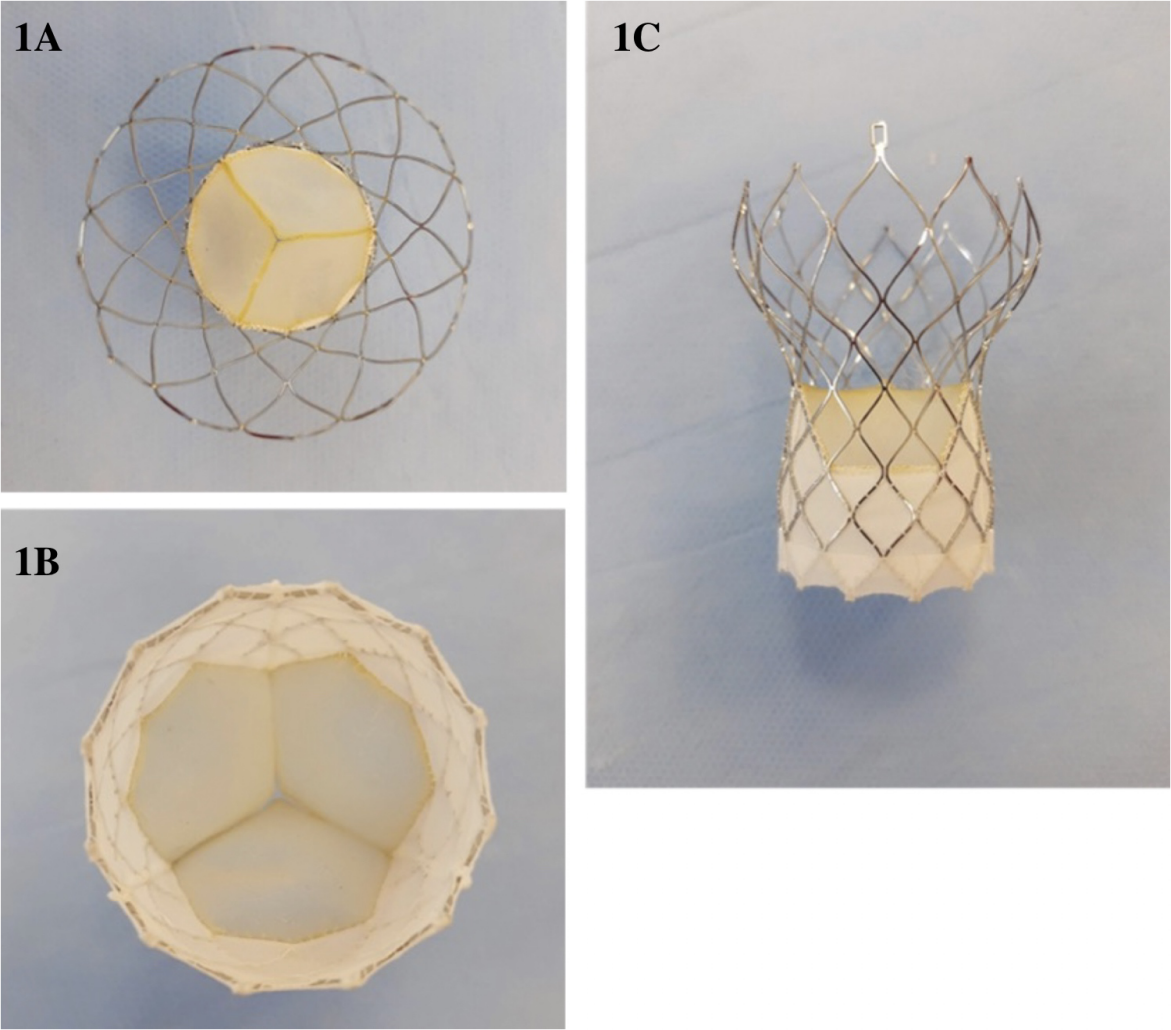
Vienna aortic valve in outflow view (**A**), inflow view (**B**) and Side view (**C**).

The Vienna Aortic Valve Self-Expandable (SE) System (P+F Products & Features GmbH, Vienna, Austria) (Figure 1) has a bovine pericardium valve with three leaflets mounted on a radiopaque nitinol self-expanding stent system designed for supra-annular positioning to optimize hemodynamic flow, and a pericardium fabric skirt on the frame (outer wrap) that covers the inflow portion of the valve to reduce paravalvular leak.

The Vienna Aortic Valve SE System enables resheathing or full recapture of the device before it is released from the Delivery System (Supplementary Figure S1). This allows the operator to reposition or remove the bioprosthesis if the implant was initially positioned too high or too low based on the native aortic valve, which reduces the risk of paravalvular leak, acute migration, and AV-conduction disturbance related to implant depth. Moreover, physicians have the option to use a lower profile introducer sheath (outer diameter) with the Delivery System, which may reduce the risk for major vascular complications (9). All valve sizes are fully pre-mounted on a single 16-F size delivery system shaft in the manufacturing facilities. This system has been integrated with a sheath of 20-F outer diameter, specifically designed to facilitate a sheathless approach for implantation in vessels with a minimum diameter of 6 mm or greater. The delivery system and sheath are made of biocompatible materials, including polymers, silicone, and metals. The distal (deployment) end of the system features an atraumatic, radiopaque tip and a capsule that covers and maintains the bioprosthesis in a crimped position. The handle, located on the proximal end of the catheter, is used to load, recapture (if necessary), and deploy the bioprosthesis.

In order to avoid the need of valve crimping and mounting during the intervention, which saves time and resources by simultaneously avoiding potential errors during these processes, the Vienna Aortic Valve is mounted on the Delivery System, ready to be used. This also allows the device to be stored in all hospitals, facilitating its use in daily clinical practice and even in emergency situations.

The system is available in four distinct sizes: 23, 26, 29, and 31 mm, which are designed to accommodate a range of aortic annulus diameters, specifically from 18 to 29 mm. For the purpose of sizing in this study, we used the perimeter-derived diameter as the key determinant. This choice was influenced by the inherent self-expandable nature of the device, which makes perimeter-derived diameters the most suitable dimension for accurate sizing (Supplementary Table S1). The Vienna TAVI device implantation target is recommended to be 3 mm below the native aortic valve annulus. There is only one Delivery System model for all valve models. The catheter can be used for femoral, subclavian/axillary, or ascending aortic (direct aortic) access sites.

### 2.2. Study design

The VIVA is a prospective, non-randomized, single-arm, single-center, first-stage FIH feasibility study, which is followed by a second-stage pivotal, multicenter, multinational study in symptomatic patients with severe aortic stenosis (SAS). The study consists of 7 visits spread out across two phases over 1 year. The clinical investigation phase ends at 30 days post-implantation and included early safety and feasibility assessments of the device after implantation in 10 symptomatic patients with SAS who were recruited in one center. The clinical investigation phase demonstrated efficient device and technical success, favorable hemodynamic performance, and a low rate of adverse events (AEs) with no mortality at 30 days. After approval of the data and safety monitoring board (DSMB), the VIVA pivotal study commenced. The subsequent 3-month, 6-month, and 1-year follow-up visits are considered post-market clinical follow-up (PMCF).

As per study protocol, changes in NYHA functional class, 6 minute walk test and 12-item Kansas City Cardiomyopathy Questionnaire (10) at screening and at 6-month follow-up, echocardiographic parameters (mean effective orifice area, mean prosthetic valve gradient, and total aortic regurgitation (AR), which included circumferential extent of AR jet(s), vena contracta, visual jet width or area, AR flow reversal in descending aorta and AR pressure half-time measurements) from baseline and at 6-month follow-up were evaluated.

As initially designed, a 4-dimensional cardiac multidetector computed tomography was performed at 3-month follow-up. The dimensions of the implanted device were measured at the inflow, valvular and outflow levels to evaluate valve eccentricity (11, 12). An eccentricity score of less than 10% (eccentricity = 1 minus minimum external valve stent diameter divided by maximum external valve stent diameter) defined a circular shape of the implanted valve (11, 12). Moreover, hypoattenuation associated with bioprosthetic leaflets, also described as hypoattenuated leaflet thickening (HALT), was evaluated with 2D axial and longitudinal multiplanar (MPR) views in diastole and 3D volume-rendered (VR) views in systole and diastole (13). Finally, in the presence of HALT, reduction of leaflet motion (RELM) was intended to be measured to define evidence of subclinical leaflet thrombosis (specifically described as hypoattenuation affecting motion, or HAM).

We report 6-month safety and performance outcomes of the first-in-human VIVA feasibility study in 10 patients diagnosed with symptomatic severe aortic stenosis.

### 2.3. Study population

Symptomatic patients with severe aortic stenosis, indicated for surgical aortic valve replacement (SAVR) with a bioprosthetic valve, were included in this study. These patients were either at an intermediate or high predicted 30-day mortality risk, as evidenced by a EuroSCORE II of 4% or higher. For patients with a EuroSCORE II below 4%, the local Heart Team evaluated their operative risk. Inclusion in the study was dependent upon the Heart Team's assessment of high operative risk due to additional existing conditions.

Relevant medical inclusion criteria were severe degenerative calcific native aortic valve stenosis assessed by TTE and characterized by aortic valve area (AVA) <1.0 cm^2^ or AVA index ≤0.6 cm^2^/m^2^ and jet velocity >4.0 m/s or mean gradient >40 mmHg; perimeter-based aortic annulus diameter between ≥18 and ≤29 mm measured by computed tomography; and symptomatic patients with NYHA class II or greater. Relevant medical exclusion criteria were congenital unicuspid or bicuspid aortic valve, severe left ventricular dysfunction with left ventricular ejection fraction (LVEF) <20% assessed by TTE, hypertrophic obstructive cardiomyopathy, pre-existing prosthetic heart valve in any position, severe mitral regurgitation, severe aortic regurgitation, or severe tricuspid regurgitation, and moderate or severe mitral stenosis. Full list of inclusion and exclusion criteria is available in the Supplementary Material.

The VIVA study was prospectively registered at ClinicalTrials.gov (NCT04861805). This study is conducted according to the Declaration of Helsinki, ICH Good clinical practice, also ISO 14155:2020, and approved by the respective institutional ethical committees. All patients provided written informed consent prior to any study procedure. Monitoring includes 100% source document verification. Echocardiographic and computed tomography (CT) analysis were performed by an independent core laboratory, and the study was supervised by an independent data safety monitoring committee, consisting of a DSMB and a medical device vigilance representative, which adjudicated all adverse events.

### 2.4. Endpoints and definitions

The objectives and endpoints were chosen based on the recommendations given by the Valve Academic Research Consortium-2 (VARC-2) for TAVIs (14).

The secondary safety, clinical and hemodynamic performance endpoints at 6 months included all-cause, cardiovascular, and non-cardiovascular mortality, cerebrovascular events (stroke and transient ischemic attack), life-threatening bleeding and conduction disturbances, requiring permanent pacemaker implantation. Moreover, re-hospitalization for valve-related complications or worsening congestive heart failure and changes in number of syncope events from baseline within 6 months were observed.

### 2.5. Statistical analysis

Statistical analyses were performed and presented using descriptive statistical methods. Continuous variables are summarized as mean ± SD. Categorical variables are summarized as frequency (percentage). Paired Student t tests were used to compare outcomes at 6 months versus baseline for echocardiographic data and 12-KCCQ scores, as well as data from pre- and post-implantation cardiac computed tomography scans to compare native vs. prosthetic valve eccentricity. Statistical significance was set at *p* < 0.05. All statistical analyses were performed using SAS version 9.4 (SAS Institute, Cary, North Carolina, USA).

## 3. Results

### 3.1. Baseline and procedural characteristics

Between November 2021 and June 2022, ten patients with symptomatic severe aortic stenosis were considered eligible for treatment with the Vienna TAVI system and were included in this first-in-human feasibility study at the Hospital of Lithuanian University of Health Sciences Kaunas Clinics. Table 1 summarizes the baseline and procedural characteristics.

**Table 1:** Baseline and procedural characteristics.

	Screening (*n* = 10)
Age, years	79 ± 5
Male	6 (60%)
Body mass index, kg/m^2^	28.2 ± 3.8
ECG	
1st degree atrioventricular block	1 (10%)
Left bundle branch block	0 (0%)
Right bundle branch block	1 (10%)
Comorbidities	
Current anemia	2 (20%)
Arterial hypertension	10 (100%)
Atrial fibrillation	2 (20%)
Benign prostatic hyperplasia	2 (20%)
Chronic lung disease	0 (0%)
Chronic renal insufficiency	10 (100%)
Stage 2 (eGFR 60–89 ml/min/1.73 m^2^)	5 (50%)
Stage 3a (eGFR 45–59 ml/min/1.73 m^2^)	3 (30%)
Stage 3b (eGFR 30–44 ml/min/1.73 m^2^)	2 (20%)
Coronary artery disease	7 (70%)
Diabetes mellitus	1 (10%)
Dyslipidemia	10 (100%)
Previous coronary artery bypass graft surgery	1 (10%)
Previous permanent pacemaker implantation	1 (10%)
Pulmonary hypertension (PA systolic pressure 31–55 mmHg)	10 (100%)
*Note: One patient may contribute to more than one category of medical condition.*	
Laboratory findings	
eGFR, ml/min/1.73 m^2^	61.2 ± 18.0
NT-pro BNP, ng/L	419.3 ± 342.1
Clinical findings and risk assessment	
EuroScore II, %	2.74 ± 1.37
High operative risk with surgical valve replacement, as assessed by the local Heart Team	9 (90%)
NYHA	2.4 ± 0.5
NYHA functional class II	6 (60%)
NYHA functional class III	4 (40%)
Left ventricular ejection fraction, %	52 ± 6
Pulmonary artery pressure, mmHg	40 ± 9
KCCQ-12, mean score	58 ± 15
6MWT, m	285 ± 97
CCS angina class 4	0 (0%)
Extracardiac arteriopathy	0 (0%)
Poor mobility	0 (0%)
Syncope events within last 12 months, *n* of patients	4 (40%)
Procedural characteristics	
Device success*	10/10 (100%)
Technical success^†^	10/10 (100%)

Data are displayed as mean ± SD, *n* (%) or n/total number of patients (%).

* Device success is defined as correct positioning of a single prosthetic investigational heart valve in the proper anatomical location AND ability to provide appropriate hemodynamics AND absence of peri-procedural mortality within 72 h after implantation.

^†^ Technical success is defined as successful vascular access, delivery AND deployment of the investigational medical device AND successful retrieval of the delivery system AND correct positioning of a single prosthetic investigational heart valve in the proper anatomical location.

6MWT, 6 min walk test; CCS, Canadian Cardiovascular Society; eGFR, estimated glomerular filtration rate; EuroScore II, European System for Cardiac Operative Risk Evaluation; KCCQ-12, 12-item Kansas City Cardiomyopathy Questionnaire; NT-proBNP, N-terminal brain natriuretic peptide precursor; NYHA, New York Heart Association; PA, pulmonary artery.

At screening, the mean age was 79 ± 5 years, 60% were men, and all patients were in NYHA functional class II or III. The mean EuroSCORE II score was 2.74 ± 1.37%, with only one patient scoring higher than 4% indicating intermediate surgical risk. The local Heart Team determined that the remaining nine patients were at high surgical risk due to comorbidities, with four of them experiencing syncope in the past 12 months. The mean aortic valve area was 0.75 ± 0.18 cm^2^ and the mean aortic valve gradient was 48.7 ± 10.8 mmHg, with a 52 ± 6% preserved mean left ventricle ejection fraction. Overall, device and technical success was achieved in all patients (100%), with no periprocedural mortality observed in any of the cases.

### 3.2. Clinical safety outcomes to 6 months

**Figure 2:**
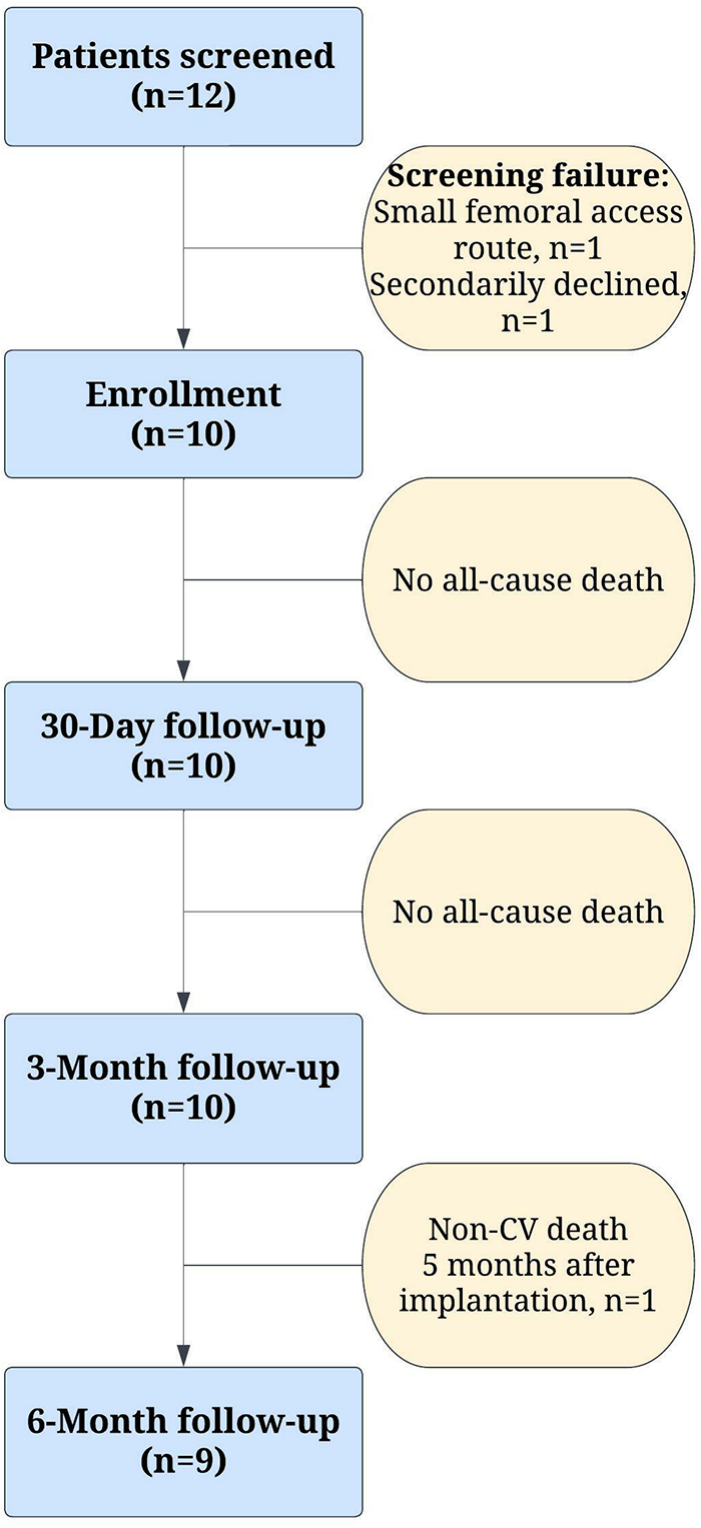
Study flow chart. Non-CV, non-cardiovascular.

Clinical outcomes at 30 days, 3 months and at 6 months are summarized in Table 2. No patients died or discontinued the study between the implantation and the 3-month follow-up period. One non-cardiovascular (non-CV) mortality unrelated to the investigational device was reported 5 months after implantation (Figure 2) due to complicated course of operation on renal oncocytoma requiring four laparotomies. Furthermore, no new cerebrovascular events, life-threatening bleeding, or conduction disturbances were observed between the 30-day and 6-month follow-ups. A total of 2 strokes occurred in 2 patients during the index hospitalization. One patient completely recovered after successful emergency mechanical thrombectomy, with no residual neurological deficit observed immediately after the procedure and at 6-month follow-up. The second patient who experienced a stroke, and who also experienced life-threatening bleeding as a result of pericardial effusion caused by the temporary wire perforation of the right ventricle, recovered only partially and required nursing care for household activities. Consequently, this patient was unable to attend the 3-month and 6-month on-site follow-up visits for the scheduled non-invasive tests. Nevertheless, this patient's clinical safety outcomes were evaluated through telephone communication. Finally, there were no re-hospitalizations for valve-related symptoms or congestive heart failure reported within 6 months.

**Table 2:** Clinical safety outcomes to 6 months.

	30-day (*n* = 10)	3-month (*n* = 10)	6-month (*n* = 9)
All-cause death	0 (0%)	0 (0%)	1 (10%)
Cardiovascular death	0 (0%)	0 (0%)	0 (0%)
Stroke	2 (20%)	0 (0%)	0 (0%)
Acute kidney injury	0 (0%)	N/A	N/A
Life-threatening bleeding*	1 (10%)	0 (0%)	0 (0%)
Hospitalization for valve-related symptoms or congestive heart failure	0 (0%)	0 (0%)	0 (0%)
Valve-related dysfunction requiring repeat procedure	0 (0%)	0 (0%)	0 (0%)
Conduction disturbances requiring permanent pacemaker implantation^†^	2 (20%)	0 (0%)	0 (0%)

Data are displayed as *n* (%).

* Pericardial effusion leading to cardiac tamponade which required pericardial drainage.

^†^ One patient with complete heart block and one patient with asymptomatic sinus bradycardia requiring permanent pacemaker implantation.

NA, not applicable.

### 3.3. Echocardiographic data

**Figure 3:**
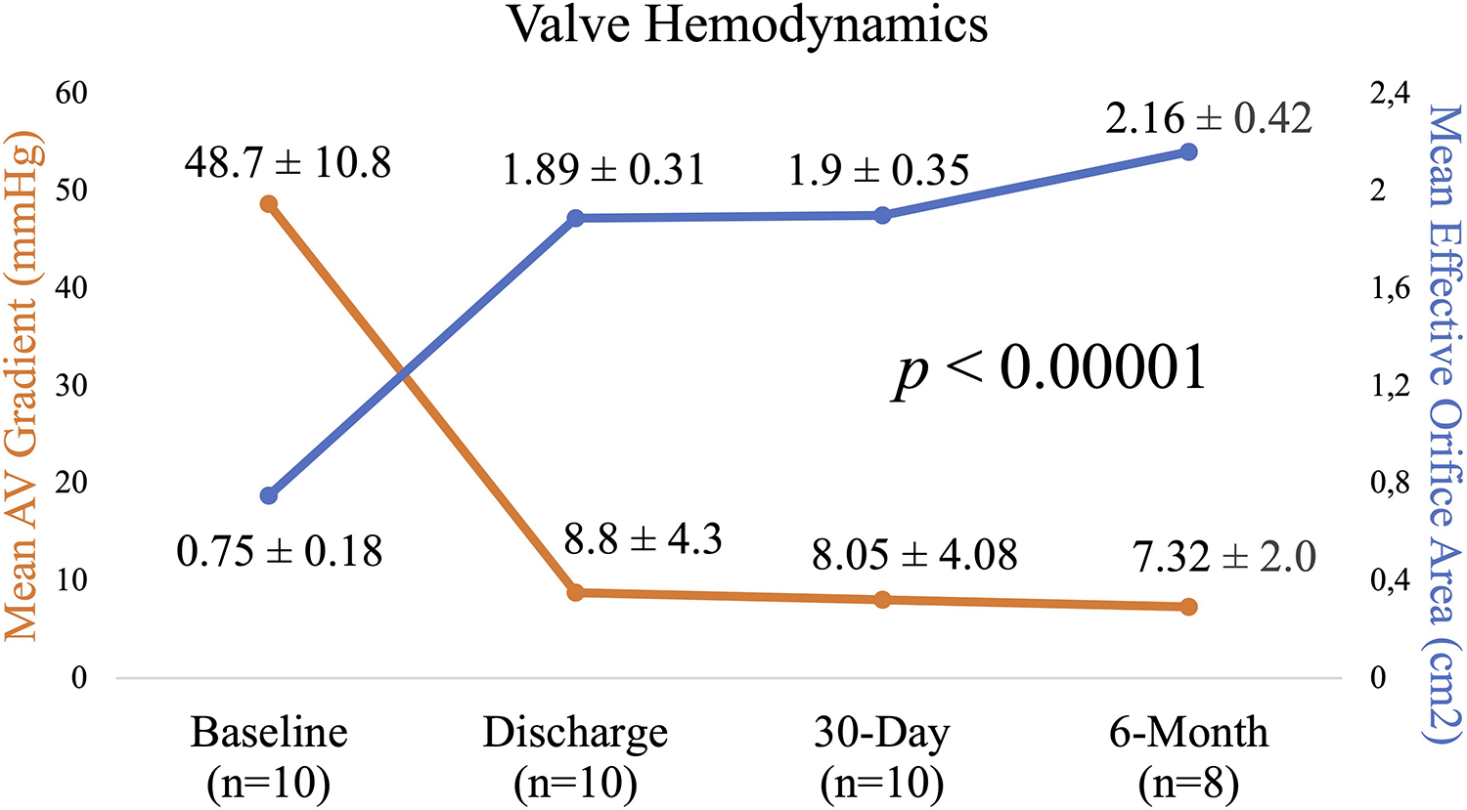
Echocardiographic parameters from baseline to 6 months: mean aortic valve gradient and mean effective orifice area.

**Figure 4:**
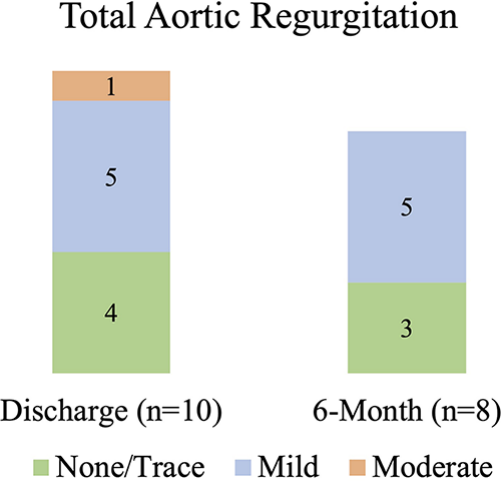
Echocardiographic parameters from discharge to 6 months: total aortic regurgitation.

At 6 months, the mean aortic valve gradient, peak velocity, aortic valve area, and effective orifice area index all significantly improved as compared to baseline (*p* < 0.00001 for all). As shown in Figure 3, the mean aortic valve gradient decreased from 48.7 ± 10.8 to 7.32 ± 2.0 mmHg and aortic valve area increased from 0.75 ± 0.18 to 2.16 ± 0.42 cm^2^. Similarly, effective orifice area index increased from 0.4 ± 0.1 to 1.13 ± 0.18 cm^2^/m^2^ and peak velocity was reduced from 4.4 ± 0.4 to 1.82 ± 0.35 m/s. As illustrated in Figure 4, there was no incidence of moderate or severe total aortic regurgitation and paralvular leak observed at 6 months. There was also no evidence of prosthetic valve dysfunction reported to 6 months.

### 3.4. Computed tomography data

8 patients underwent post-implantation 4-dimensional cardiac multidetector computed tomography at 3-month follow-up. One patient with a history of stage 3b chronic renal insufficiency did not undergo a CT scan due to borderline eGFR laboratory values, and the second patient was not able to attend the scheduled visit due to a persistent neurological deficit after stroke. Native aortic annular eccentricity pre-TAVI was greater than transcatheter bioprosthetic heart valve (THV) inflow eccentricity (22.3 ± 7.3% vs. 10.4 ± 6.5%, *p* < 0.00001). The eccentricity of the THV was generally greater at the inflow (10.4 ± 6.5%) and valvular level (9.8 ± 4.9%), and lowest at the outflow (1.9 ± 1.3%). THV circularity was observed in seven valves at the inflow and valvular levels, and eight valves at the outflow level. Moreover, one of eight post-implant CT scans was inconclusive for assessing HALT due to the inability to see the leaflet coaptation. On a 2-dimensional MPR diastolic phase and 3-dimensional volume-rendered projection in systole and diastole, leaflet coaptation with no HALT was reported in the other seven good-quality post-implant CT scans.

### 3.5. Quality-of-life and functional status

**Figure 5:**
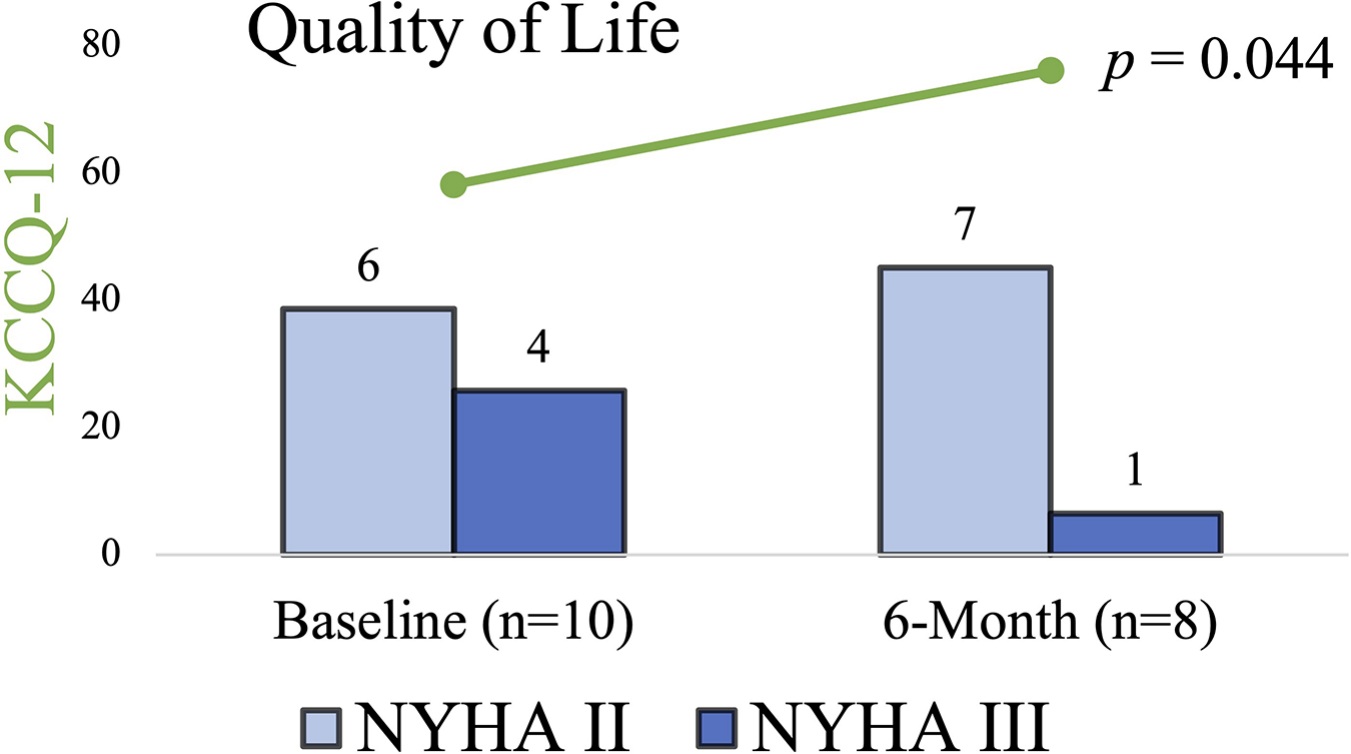
12-KCCQ mean score and NYHA classification at baseline and 6 months. In the Quality-of-Life questionnaires the patients reported a significant improvement, also NYHA functional class improved in two patients, remained unchanged in one patient, was not reported in one patient due to prolonged recovery, and one patient died due to non-cardiovascular cause. KCCQ-12, 12-item Kansas City Cardiomyopathy Questionnaire; NYHA, New York Heart Association.

As shown in Figure 5, in the Quality-of-Life questionnaires, the patients reported a significant improvement from the baseline mean score of 58 ± 15 to 76 ± 20 at 6 months based on the 12-item Kansas City Cardiomyopathy Questionnaire (*p* = 0.044). Out of nine patients, the NYHA functional class improved in two, remained unchanged in one, and was not reported in one. At 6 months, there was an increase in mean 6 min walk distance from 285 ± 97 to 347 ± 57 m (*p* = 0.073) and a decrease in NT-proBNP laboratory levels from 419.25 ± 342.07 to 227 ± 165.95 ng/L (*p* = 0.144), respectively. Eventually, there were no syncope events reported within 6 months of the procedure.

## 4. Discussion

First-in-human experience with the Vienna Self-Expandable Aortic Valve system shows favorable and continued clinical safety and effectiveness profile of this novel TAVI system for the treatment of severe aortic stenosis in intermediate and high-risk patients.

### 4.1. Safety

The results of this study demonstrated the clinical safety of the device, with a reported all-cause mortality of 10% and a 0% cardiovascular mortality rate within a 6-month period. Stroke remains a major risk in the TAVI population (14, 15). The reported incidence of disabling stroke at 6 months is consistent with findings from other TAVR studies in high-risk patient populations (16, 17). Given the limited number of disabling strokes and the small sample size, our study did not have the statistical power to identify stroke predictors. However, it is worth noting that one patient experienced a disabling acute stroke post-procedure. This patient had a history of permanent atrial fibrillation and was on a new oral anticoagulant therapy regime. Prior to the TAVI procedure, aortic valve calcification was assessed using non-contrast electrocardiogram-gated computer tomography scans with the Agatston method. This revealed a score of 2,400 AU, suggesting severe calcification. As a result, the patient required two pre-dilatations with valvuloplasty balloons to aid the expansion of the calcified leaflets, potentially heightening the risk of stroke (18). Finally, it should be emphasized that valve recapture and repositioning were not necessary for this patient, as the optimal valve position was obtained on the first attempt.

Within the scope of this study, conduction disorders necessitated periprocedural PPM implantation in 2 out of 10 patients. One of these patients developed a third-degree atrioventricular block post-implantation, a finding that aligns with other TAVI studies (19). This particular case was the first attempted Vienna valve implantation. Due to the operator's first experience with the Vienna TAVI system, the valve was implanted deeper into the LVOT than the recommended nominal target of 3 mm below the aortic annulus, as seen on fluoroscopy. A 3-month post-procedure CT scan showed the valve stent frame positioned 6 mm below the aortic annulus, a discrepancy that could potentially heighten the risk of conduction disturbances (19). However, no increase in mitral valve gradients or more than mild PVL was observed for this patient during a 6-month cardiac ultrasound follow-up.

In a broader perspective, permanent pacemaker implantation rates after TAVI have ranged from 3% to 40%, with balloon-expandable valves being associated with lower implantation rates than self-expandable valves (19). Finally, since this was a first-in-human experience associated with an operator's early learning curve, the average reported PPM rates may be reduced after gaining more expertise with the Vienna TAVI system.

### 4.2. Hemodynamic performance

Significant PVL after valve implantation has been linked to higher morbidity and mortality (20). The Vienna valve demonstrated low rates of total aortic regurgitation up to 6 months, with none of the patients having moderate to severe regurgitation. There were no cases of central regurgitation, implying that leaflet coaptation is adequate up to 6 months and that all total aortic regurgitation is paravalvular. No moderate or severe PVL was noted and may be attributed to the appropriate valve frame geometry and an external pericardial skirt designed for leak prevention. It should also be noted that none of the patients in this study received post-dilation because effective annulus sealing was ensured.

In addition, adequate oversizing and full prostheses expansion allows for enough radial force to be applied to secure the THV, assure effective sealing, reduce PVL (21), and may decrease the occurrence of HALT (22). With self-expandable valves, some degree of underexpansion at the inflow level is to be expected (22). Based on the post-TAVI CT data at 3-month follow-up, the THV expansion completeness and residual eccentricity of the Vienna device appeared to be in line with that of the self-expandable CoreValve device (12).

Moreover, the echocardiographic results at 6-month follow-up also showed the prosthesis to be effective in terms of hemodynamic parameters, with significant improvements in aortic valve area, mean gradient, and peak velocity compared to baseline values. These outcomes are similar to those of balloon-expandable aortic devices of the first and second generations, as well as other commercially available nitinol valves (23). Respectively, no evidence of subclinical leaflet thrombosis was reported on 3-month CT data. Interestingly, prior studies have also demonstrated that self-expanding THV (6%–8%) has a lower incidence of HALT than balloon-expandable Sapien S3 THV (14%–28%) (24, 25). Notably, two of the seven patients with analyzable, good-quality CT scans were on long-term anticoagulation therapy with new oral anticoagulants due to persistent atrial fibrillation.

### 4.3. Functional status and quality-of-life

Recent studies have shown that patients who undergo transcatheter aortic valve implantation experience significant improvements in their quality of life up to 30 days, 6 months, and 1 year after the procedure (26). The results of the current study are consistent with these observations, with patients reporting significant improvements in their health status and quality of life at the 6-month follow-up. Only two patients in the study experienced an improvement in their NYHA functional class at 6 months, improving from class III to class II. It is important to note that none of the patients in the study qualified for the NYHA class IV at the screening and would have potentially seen even more significant benefits from the procedure (27). Additionally, the mean 6 minute walk performance of patients improved by 22%, which is in line with earlier studies that have shown improvements in 6 minute walk performance after TAVI, likely reflecting a clinically important improvement in functional status (28).

### 4.4. Limitations

The current study is based on data collected from a single center and the patient population is relatively small, which is a limitation. Furthermore, not all patients participated in the follow-up visit, which makes it difficult to analyze hard clinical endpoints. Therefore, it is important to note that the results of the study need to be confirmed in larger studies with a larger patient population. Another limitation of the study is that it is non-randomized, and does not provide a direct comparison with other TAVI valves. Therefore, it is not possible to draw definitive conclusions about the performance and durability of this new valve compared to existing devices. To gain a better understanding of the valve's performance, long-term results beyond 6 months are needed to make final statements about the valve's durability and performance.

## 5. Conclusions

The VIVA first-in-human feasibility trial findings demonstrate that the self-expandable Vienna bioprosthetic aortic valve has a favourable and sustained 6 month safety and performance outcomes in patients with symptomatic severe aortic stenosis who are at intermediate or high surgical risk. The second phase of the VIVA pivotal trial has already commenced, with a larger patient population evaluating long-term safety and clinical efficacy of this novel device.

## Data Availability

The datasets presented in the article are not readily available because licensing restrictions applied to the current study. Requests to access the datasets should be directed to the corresponding author.
