# Supplementary material for: Safety and performance of the Vienna self-expandable transcatheter aortic valve system: 6-month results of the VIVA first-in-human feasibility study
Source: Front Cardiovasc Med. 2023 Jul 13;10:1199047. doi: 10.3389/fcvm.2023.1199047 (PMC10373888; doi:10.3389/fcvm.2023.1199047)
Supplement: Supplementary file 1 [file Datasheet1.docx]

**SUPPLEMENTARY MATERIAL**

**Patient inclusion and exclusion criteria for the VIVA FIH study**

**Inclusion criteria**

1. Male and Female.
2. Age ≥ 65 years at time of consent.
3. Women of non-childbearing potential.
4. Severe degenerative calcific native aortic valve stenosis with the following criteria assessed either by resting or dobutamine stress TTE:
   - 1. Aortic valve area (AVA) < 1.0 cm2 or AVA index ≤ 0.6 cm2/m2 and
     2. Jet velocity > 4.0 m/s or mean gradient > 40 mmHg.
5. Symptomatic aortic stenosis (AS), defined as a history of at least one of the following:
   1. Dyspnea that qualifies at NYHA class II or greater.
   2. Angina pectoris.
   3. Cardiac syncope.
6. Subject is considered at intermediate or high risk for surgical valve replacement based on at least one of the following:
   1. EuroSCORE II ≥ 4%.
   2. Agreement by the Heart Team that subject is at high operative risk of serious morbidity or mortality with surgical valve replacement.
7. The local Heart Team, including at least 1 cardiothoracic surgeon and 1 interventional cardiologist, deems the patient to be eligible for transfemoral TAVI.
8. Perimeter-based aortic annulus diameter between ≥ 18 and ≤ 29 mm measured by computed tomography (CT) performed within 90 days prior to planned implantation.
9. Adequate iliofemoral access with minimum average vessel diameter of ≥ 6.0mm and acceptable level of vessel calcification and tortuosity for safe placement of the introducer sheath.
10. The distance from coronary ostia to aortic anulus > 12 mm.
11. Patient (or legal representative) understands the study requirements and the treatment procedures and provides written informed consent.
12. The patient and the treating physician agree that the patient will return for all required post-procedure follow-up visits.

**Exclusion criteria**

**Cardiovascular system:**

1. Patient has a congenital unicuspid or bicuspid aortic valve or non-calcified valves.
2. Evidence of an acute myocardial infarction (MI) ≤ 30 days before the IMD implantation (defined as Q-wave MI or non- Q-wave MI with total CK elevation ≥ twice normal in the presence of CK-MB elevation and/or troponin elevation).
3. Patient has had a cerebrovascular stroke or TIA within the past 90 days before IMD implantation.
4. Patient has a hypertrophic obstructive cardiomyopathy.
5. History of any therapeutic invasive cardiac procedure (including balloon aortic valvuloplasty) within 30 days prior to the planned IMD implantation (except for pacemaker implantation which is allowed).
6. Untreated clinically significant coronary artery disease requiring revascularization at the screening visit.
7. Severe left ventricular dysfunction with left ventricular ejection fraction (LVEF) < 20% by echocardiography, contrast ventriculography, or radionuclide ventriculography within 90 days prior.
8. Patient with cardiogenic shock manifested by low cardiac output and hemodynamic instability and vasopressor dependence, or mechanical hemodynamic support.
9. Patients with clinically significant conduction abnormalities (clinically significant sinus bradycardia, sinus block or pauses, clinically significant atrioventricular (AV)-block >I) at screening and at time of valve implantation.
10. Patient has severe peripheral vascular disease:
    1. including aortic aneurysm defined as maximal luminal diameter > 5 cm or with documented presence of thrombus, marked tortuosity, narrowing of the abdominal aorta, severe unfolding of the thoracic aorta or thick (> 5 mm), protruding or ulcerated atheroma in the aortic arch or
    2. symptomatic carotid or vertebral disease or successful treatment of carotid stenosis within 30 days before IMD implantation.
11. Patient with iliofemoral vessel characteristics that would preclude safe passage of the introducer (severe calcification, tortuosity (> two 90-degree bends), diameter < 6mm, or subject has had an aorto-femoral bypass).
12. Patient with active bacterial endocarditis within 6 months of planned IMD.
13. Patient has (echocardiographic/ CT and/or MRI) evidence of intra-cardiac mass, thrombus or vegetation.
14. Patient has a pre-existing prosthetic heart valve in any position (Note: mitral ring is not an exclusion).
15. Patient has severe mitral regurgitation, severe aortic regurgitation or severe tricuspid regurgitation, moderate or severe mitral stenosis.
16. Patient has a need for emergency surgery for any reason at time of screening and valve implantation.

**General:**

1. Any condition considered a contraindication for placement of a bioprosthetic valve (e.g. patient with contraindication to oral antiplatelet therapy).
2. Patient with renal insufficiency (eGFR < 30 ml/min per the Cockcroft-Gault formula) and/ or renal replacement therapy and/ or has serum creatinine level > 3.0 mg/dL or 265 μmol/L replacement therapy at the time of screening.
3. Patient with significant pulmonary disease (FEV1 < 30%) or currently on home oxygen.
4. Severe pulmonary hypertension (e.g., PA systolic pressure / systemic pressure >1 or mean pulmonary pressure > 55 mmHg assessed by echocardiography).
5. Patients with evidence of an active systemic infection or sepsis.
6. Patient has a known hypersensitivity or contraindication to contrast media, bovine tissue, nitinol (titanium or nickel), contraindication to oral antiplatelet therapy (aspirin, ticlopidine or clopidogrel) or heparin.
7. Patient has a hemoglobin < 9 g/dL, platelet count < 50,000 cells/mm^3^ or > 700.000 cells/mm^3^, or white blood cell count < 1.000 cells/mm^3^, history of bleeding diathesis or coagulopathy.
8. Patient has peptic ulcer disease or history of gastrointestinal bleeding within the past 3 months.
9. Patient refuses blood transfusions.
10. Patient has a life expectancy of less than 12 months due to non-cardiac, co-morbid conditions based on the assessment of the investigator at the time of enrolment.
11. Patient is pregnant or breastfeeding.
12. Severe dementia (resulting in either inability to provide informed consent for the study/procedure, prevents independent lifestyle outside of a chronic care facility, or will fundamentally complicate rehabilitation from the procedure or compliance with follow-up visits).
13. Other medical, social, or psychological conditions that in the opinion of the Investigator precludes the patient from appropriate consent or adherence to the protocol required follow-up exams.
14. Patient is currently participating in another investigational drug or device study that has not reached its primary endpoint (excluding observational studies).

**Supplementary Table 1.** Vienna Aortic Valve specifications.

| **Vienna aortic valve model** | **A range of aortic annulus diameters** | **Proximal diameter** | **Distal diameter** | **Frame height after deployment** |
| --- | --- | --- | --- | --- |
| **23** | 18 – 20 mm | 23 mm | 34 mm | 45 mm |
| **26** | 20 – 23 mm | 26 mm | 40 mm | 55 mm |
| **29** | 23 – 26 mm | 29 mm | 42 mm | 51 mm |
| **31** | 26 – 29 mm | 31 mm | 43 mm | 52 mm |

**Supplementary Figure 1.** Delivery system of the Vienna Aortic Valve.
