# Supplementary figures and images for: Safety and performance of the Vienna self-expandable transcatheter aortic valve system: 6-month results of the VIVA first-in-human feasibility study
Source: Front Cardiovasc Med. 2023 Jul 13;10:1199047. doi: 10.3389/fcvm.2023.1199047 (PMC10373888; doi:10.3389/fcvm.2023.1199047)

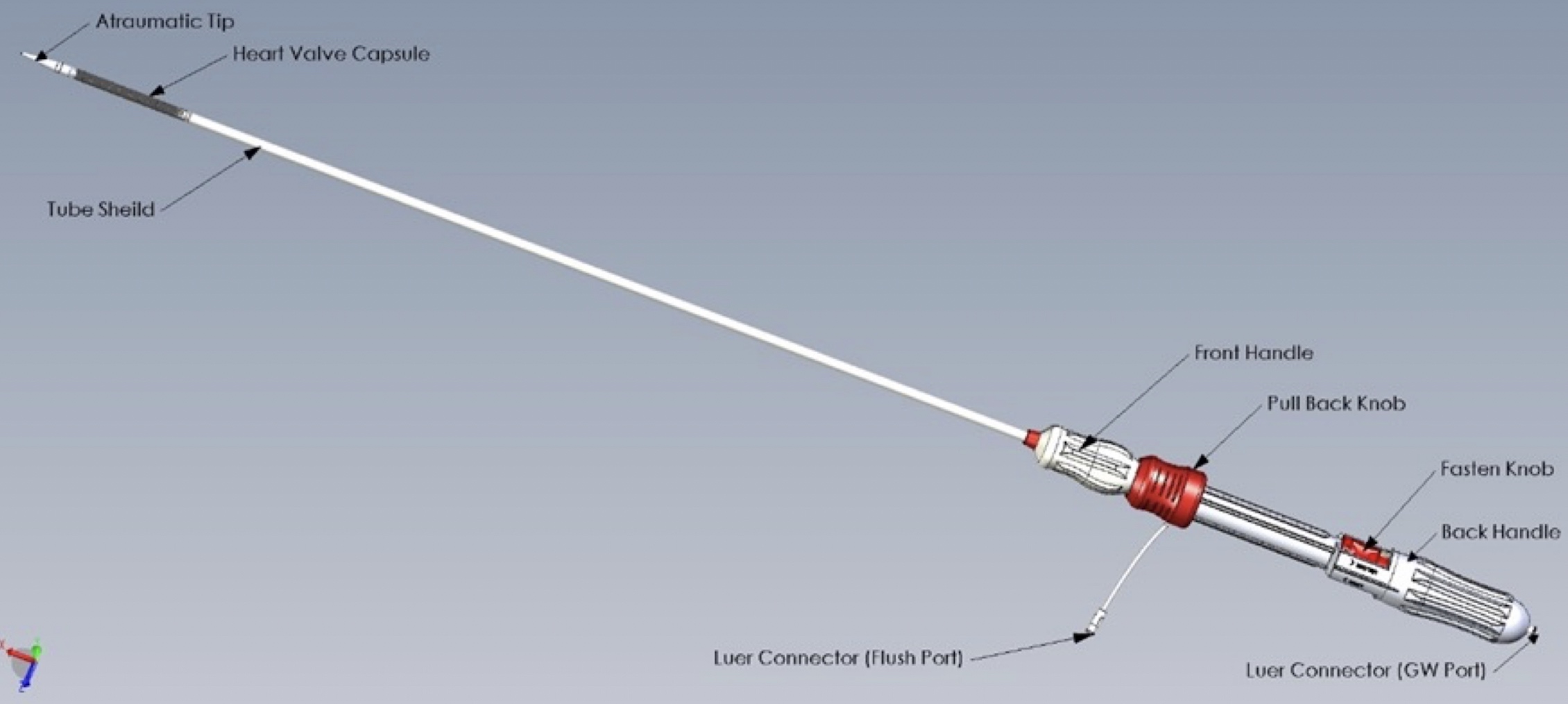

Supplement: Supplementary file 2 [file Image1.jpeg]
